# Augmented Bayesian Data Selection: Improving Machine Learning Predictions of Bragg Grating Spectra

**DOI:** 10.3390/s25164970

**Published:** 2025-08-11

**Authors:** Igor Nechepurenko, M. R. Mahani, Yasmin Rahimof, Andreas Wicht

**Affiliations:** Ferdinand-Braun-Institut (FBH), Gustav-Kirchhoff-Straße 4, 12489 Berlin, Germany

**Keywords:** machine learning, bragg gratings, spectral analysis, bayesian optimization

## Abstract

Bragg gratings are fundamental components in a wide range of sensing applications due to their high sensitivity and tunability. In this work, we present an augmented Bayesian approach for efficiently acquiring limited but highly informative training data for machine learning models in the design and simulation of Bragg grating sensors. Our method integrates a distance-based diversity criterion with Bayesian optimization to identify and prioritize the most informative design points. Specifically, when multiple candidates exhibit similar acquisition values, the algorithm selects the point that is farthest from the existing dataset to enhance diversity and coverage. We apply this strategy to the Bragg grating design space, where various analytical functions are fitted to the optical response. To assess the influence of output complexity on model performance, we compare different fit functions, including polynomial models of varying orders and Gaussian functions. Results demonstrate that emphasizing output diversity during the initial stages of data acquisition significantly improves performance, especially for complex optical responses. This approach offers a scalable and efficient framework for generating high-quality simulation data in data-scarce scenarios, with direct implications for the design and optimization of next-generation Bragg grating-based sensors.

## 1. Introduction

Bragg gratings are periodic structures that enable precise control of light propagation in optical systems [1,2,3,4,5]. They function by selectively reflecting specific wavelengths while allowing others to pass through, making them fundamental components in optical fiber communication [6], sensing [7,8], and laser stabilization [9]. Their ability to manipulate spectral properties with high resolution has led to widespread use in fields such as telecommunications [10], biomedical sensing [11,12,13,14], and industrial monitoring [15,16,17,18,19,20]. However, optimizing the design and spectral response of Bragg gratings remains a computationally intensive challenge, requiring advanced modeling techniques to predict and tailor their behavior effectively [21,22,23,24].

Despite their versatility, predicting the spectral response of Bragg gratings is complex due to the interplay of multiple parameters and complex multi-mode interactions. Traditional approaches, such as the transfer matrix method and coupled mode theory [25,26,27,28,29,30,31,32,33], offer computationally efficient but less accurate methods. The challenge is intensified by the need to balance accuracy and computational efficiency, making it difficult to explore a diverse range of grating structures without excessive computational costs.

Inverse design [34,35,36,37,38] and machine learning (ML) for modeling and predicting the spectral response of Bragg gratings [39,40,41] are promising alternatives. By training models on precomputed datasets, ML techniques can rapidly approximate spectral characteristics, significantly reducing computational overhead [42,43,44]. Deep learning and regression-based models have shown potential in capturing the nonlinear relationships between design parameters and spectral outputs, providing a data-driven approach to spectrum prediction and evaluation [45,46,47,48]. However, the effectiveness of these models heavily depends on the quality and diversity of the training data, highlighting the importance of selecting representative samples that accurately span the parameter space [49].

Traditional data selection methods, such as uniform sampling or random selection, often fail to provide an optimal dataset for training ML models, especially in high-dimensional parameter spaces. These approaches may yield redundant data points, increasing computational costs [50,51]. Moreover, they may overlook critical spectral variations that arise from complex interactions between grating parameters. As a result, there is a need for more intelligent data selection strategies that ensure comprehensive coverage of the design space while minimizing computational costs.

To address these limitations, this study proposes integrating Bayesian Optimization (BO) with distance-based criteria for selecting training data more effectively for Bragg grating spectrum prediction. BO employs probabilistic surrogate models to efficiently explore the design space, prioritizing data points that are expected to yield the greatest improvements in model accuracy [49,52,53]. When combined with distance-based sampling, this approach ensures a more diverse and representative dataset, leading to more robust and generalizable machine learning (ML) models.

BO has previously been applied to Bragg grating design, leveraging surrogate models—typically Gaussian Processes—to navigate high-dimensional parameter spaces while reducing the number of costly simulations [15]. Prior studies have demonstrated its effectiveness in optimizing grating parameters such as chirp profiles, apodization, and segmentation for various photonic applications [54,55,56].

Building on these developments, this work introduces a systematic framework for optimizing training data selection in ML-based Bragg grating models. By combining BO with distance-based sampling, the method aims to improve prediction accuracy while minimizing the training dataset. Comparative evaluations against conventional data selection strategies highlight the efficiency and effectiveness of the proposed approach, contributing to the advancement of data-driven optical modeling for integrated photonics.

## 2. Materials and Methods

**Figure 1:**
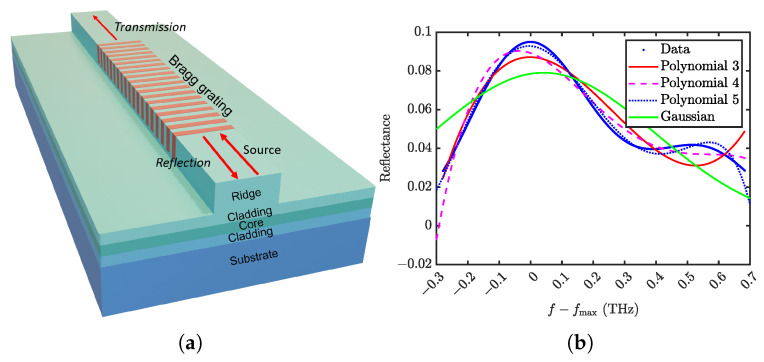
(**a**) Ridgewaveguide with the surface Bragg grating. (**b**) Example reflection spectrum fitted with various fit functions.

To evaluate our approach, we start by generating an initial dataset in the Bragg grating design space using a uniform mesh grid to ensure a comprehensive representation of the design variables. For the investigation, we choose a ridge waveguide with surface Bragg gratings (Figure 1a). The structure was designed in a GaAs material platform, known for its excellent electro-optical properties. However, it is applied in other material platforms as well.

To generate a comprehensive dataset for analyzing the optical performance of GaAs ridge waveguides with integrated Bragg gratings, a specific device geometry was designed and simulated. The waveguide features a ridge width and height of 5 μm and 1.4 μm, respectively, tailored for single-mode operation. On top of this ridge lies a higher-order rectangular Bragg grating of varying length. These features allow precise tuning of the structure’s optical reflectance, which is centered near 2.8 × 10^14^ Hz (wavelength ≈ 1064 nm), making it highly suitable for specific laser applications. We generated a database of the reflectance spectra by varying the parameters, namely, length of Bragg grating (25–75 μm), depth (1.1–1.6 μm), width (0.025–0.12 μm) and refractive index of gratings’ grooves (1.95–2.9), chirp value (relative period variation along the grating ±0.001) and the order of the grating (5–9). Each of the parameters was sampled with 5 linearly spaced points.

To accurately model and simulate the interaction of light with this complex structure, the Lumerical FDTD solver was integrated with MATLAB R2022a for automated simulation control (see [22,23,32] for more details). A uniform mesh size of 20 nm was employed across all dimensions, providing high spatial resolution with approximately one billion mesh points per simulation. A total of 15,625 simulations were conducted, resulting in a dataset that spans a technologically feasible range of parameters.

After completing the simulations, mode projection was used to compute reflectance values, and the central lobe of the reflectance spectra was analyzed using Gaussian fitting techniques. Due to the shallow groove depths and high duty cycles used, radiation losses were minimal, and the central reflection lobe remained symmetric. This allowed for an accurate Gaussian approximation of the central lobe using data points exceeding one-third of the peak amplitude. The fitting achieved over 99% accuracy, validating the use of this method for analyzing the amplitude and bandwidth dependence on the Bragg grating’s physical parameters. This dataset provides a reliable foundation for optimizing ridge waveguide Bragg gratings in high-performance photonic devices.

## 3. XGBoost-Based Prediction of Bragg Spectra

### 3.1. Data Generation Fit Comparison: Gaussian vs. Polynomial

To reduce the dimensionality of the output data, we fit the reflectance spectra with various functions and use the fit parameters as the output features in our database (Figure 1b). As a benchmark, we use a simple fit of the top two-thirds of the central lobe in reflectance spectra with a Gaussian function. It was previously demonstrated that such an approach is efficient for describing the amplitude and bandwidth of the central lobe of the Bragg spectrum [43].

Due to the length variation of the Bragg gratings in the database, the bandwidth of the Bragg reflectivity peak varies significantly. As a result, describing the spectrum in the same bandwidth might cause low precision for narrow spectra (longer structures) or negligence of the spectral features for broad spectra (shorter structures). To avoid this, two approaches are generally possible to recalculate bandwidth for each spectrum: (i) considering the fixed bandwidth for each length, (ii) automatically recalculating the bandwidth depending on the shape of the central lobe. The bandwidth of the Bragg reflectance of the weak grating is inversely proportional to its length. Thus, one can identify the base bandwidth for a fixed length and recalculate it for other cases. Such an approach would be efficient for mostly symmetric spectra. The second approach requires an automatic determination of the bandwidth for the point above a threshold reflectivity, but it can be more flexible and applicable for complex spectra. We applied the second approach in the text to adapt to the asymmetry of some of the spectra. Those spectra are observed for chirped gratings and structures with deep grooves, causing high radiation losses.

The results of the comparison of the Gaussian and polynomial fitting methods are presented in Table 1. The fit performed by different methods was applied to every spectrum in the database. To evaluate the accuracy of the fit, we use the coefficient of determination ($R^{2}$) and root mean squared error (RMSE) metrics. As expected, a polynomial function of the 5th order demonstrates the highest precision. In particular, the presence of additional peaks in some of the Bragg spectra (Figure 1b) requires higher-order polynomials to efficiently fit the top of the spectra and their asymmetrical features. The produced dataset of coefficients for various simulations is available here: Ref. [57].

### 3.2. ML Performance: Parameter Prediction vs. Curve Prediction

Once the fit coefficients for every fit function are calculated, we consider the problem of predicting the spectrum. By reframing the problem as a regression task, we use machine learning to train and predict the fit parameters of the spectra and evaluate the performance using two approaches. The first approach, parameter prediction, directly compares the predicted fit parameters with the true fit parameters. The second approach, curve prediction, assesses the accuracy of the entire reconstructed spectra. After predicting the fit parameters with machine learning, we regenerate the reflectance spectra using both the predicted and true parameters. These reconstructed spectra are then compared, providing an evaluation of how well the model captures the shape and features of the spectra.

To solve the regression problem with limited data, we apply extreme gradient boosting (XGBoost) technique, a data-efficient machine learning algorithm, to predict the parameters of interest [58]. The XGBoost method is a highly efficient implementation of gradient-boosted decision trees, which performs well in regression and classification tasks. Its core principle involves iteratively constructing a series of decision trees, where each subsequent tree minimizes the errors of its predecessor by focusing on the residuals. Unlike standard gradient boosting, XGBoost incorporates several enhancements such as regularization to reduce over-fitting, a sparsity-aware algorithm for better handling of missing data, and parallelized tree construction for improved scalability [59,60,61]. In this study, XGBoost was utilized to predict fit parameters, achieving high precision due to its ability to model non-linear relationships and adapt to varying complexities of spectral features. The chosen configuration included the use of the ’dart’ booster, which introduces dropout techniques into the boosting process to improve generalization. The learning rate is set to 0.08 to balance training speed and convergence stability. A maximum tree depth of 8 allows the model to capture moderately complex interactions in the data, while the number of estimators is fixed at 170 to control the model’s capacity.

The selection of XGBoost is motivated by the limited availability of data. While neural network-based techniques offer high efficiency and flexibility, they typically require large amounts of training data, which is not feasible when simulations are performed with high precision [41,45]. On the other hand, simpler methods such as k-NN or linear regression require less data but fail to achieve the necessary accuracy [43]. As a result, XGBoost provides a well-balanced compromise for analyzing limited simulation data obtained from highly accurate FDTD methods.

We apply XGBoost to our databases of fit coefficients (Table 2). To evaluate the accuracy of the prediction, we use the coefficient of determination ($R^{2}$), root mean squared error (RMSE) metrics, and mean absolute percentage error (MAPE). The dataset Ref. [57] for each fit method was randomly split into 90% training and 10% test subsets. This process was repeated 100 times to ensure statistical robustness, and the average values along with standard deviations of the evaluation metrics were computed and reported. We can observe the overall decrease in the precision of prediction for all the evaluation methods with an increase in the polynomial order.

In Table 3, we compare the precision of the predicted coefficients for the fit functions. We also compare the accuracy of curve prediction, which involves comparing the reconstructed spectra to the true spectra obtained from the FDTD simulation method. Predicting the Gaussian fit parameters exhibited higher precision, due to its simplicity and fewer parameters, achieving an average mean squared error, $MSE=4.94\times 10^{-6}$. However, their oversimplified representation of spectral features, such as asymmetry and radiation losses, resulted in lower accuracy for curve prediction $MSE=6.49\times 10^{-6}$.

**Figure 2:**
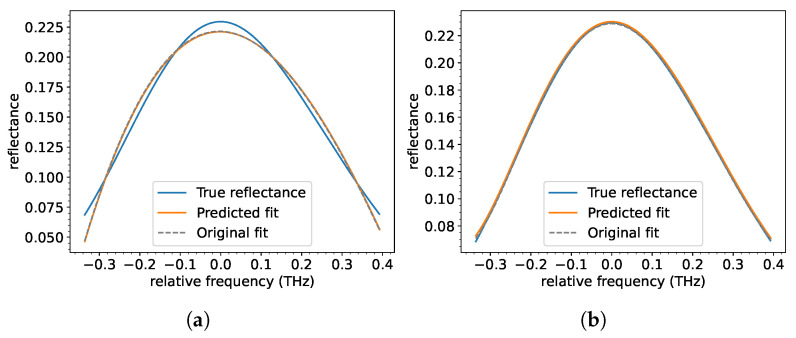
True and predicted fit for a spectrum performed with a polynomial function of the (**a**) 3rd and (**b**) 5th order. Relative frequency here is defined as the difference between the actual and the reference frequency (Bragg resonance frequency).

In contrast, polynomial fits, especially of higher orders (e.g., n=5), provided a more detailed representation of the spectra, capturing critical features like side peaks and asymmetries (Figure 2b). This resulted in a superior curve prediction accuracy despite slightly reduced precision for parameter prediction. These results underscore the trade-off between computational simplicity and spectral-prediction accuracy. The analysis highlights XGBoost’s suitability for tasks requiring detailed spectral reconstruction.

In summary, XGBoost effectively balances parameter prediction accuracy and curve reconstruction precision, demonstrating adaptability to complex spectral features. Its efficiency highlights its potential for optimizing machine learning workflows in optical system simulations. These insights lay the foundation for applying advanced data selection strategies, explored further in Section 3, to enhance data acquisition efficiency and model performance.

## 4. Data Acquisition

### 4.1. Bayesian Optimization for Database Generation

Similar to the previous analysis, we implement a Bayesian-based sampling (BBS) approach [49] to select the most informative data points for the best curve prediction. In this approach, Gaussian process regression identifies data points with maximal uncertainty, building minimal yet highly informative datasets. The approach was applied to Bragg grating designs, where the Bayesian-based database required significantly fewer data points compared to traditional uniform and random sampling methods while maintaining the same accuracy. BBS has been shown to reduce data requirements by an order of magnitude, highlighting its efficiency, particularly in resource-intensive simulations like FDTD. This methodology not only ensures high accuracy in predictions performed by ML but also demonstrates its broader potential for data-driven modeling across scientific disciplines, enabling cost-effective and precise database generation for ML applications.

In order to avoid dependency on initial conditions in the BBS method, we averaged $R^{2}$ values for five different initial conditions. We also averaged the outcomes on randomly selected data points 10 times. The efficiency was calculated for different fit methods. To do that, we took the maximum prediction precision achieved with the random database and calculated the data points needed to achieve the same precision with BBS.

**Figure 3:**
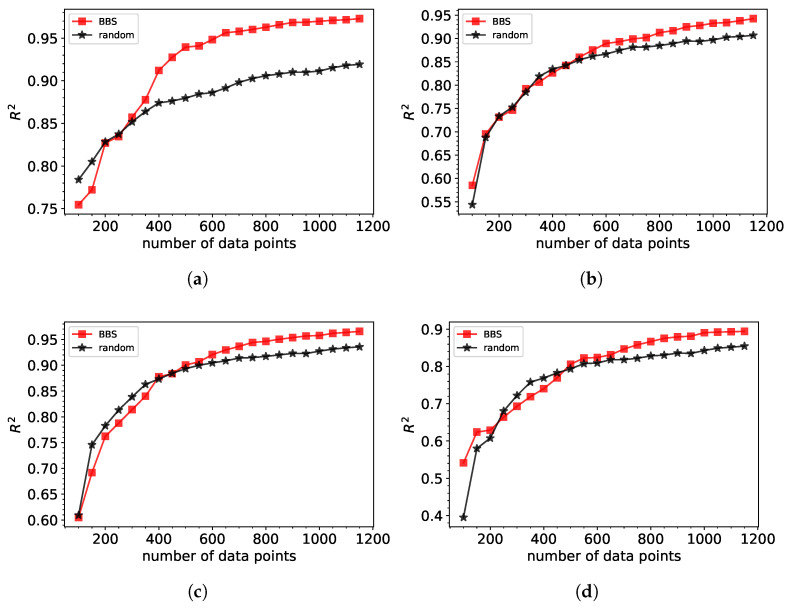
Performance of XGBoost machine learning model trained on databases generated by BBS versus random selection of points for (**a**) Gaussian fit (**b**) polynomial of 3rd order (**c**) polynomial of 4th order (**d**) polynomial 5th order.

Figure 3 compares the performance of machine learning models trained on datasets generated using BBS and those created via random sampling. The results demonstrate that the BBS method significantly outperforms random sampling in terms of predictive accuracy, as measured by the ($R^{2}$), for both Gaussian and polynomial fits. Databases constructed through BBS achieve similar $R^{2}$ values with much fewer data points, emphasizing the efficiency of the BBS-selected data.

For Gaussian fits with three parameters, the BBS approach reaches an $R^{2}$ of approximately 0.95 using about 600 data points, whereas random sampling requires considerably more data to attain comparable accuracy. Similarly, for polynomial fits of the 3rd and 4th orders, BBS demonstrates its ability to handle increasing spectral complexity, consistently achieving better predictions with reduced dataset sizes. In the case of the 4th-order polynomial fit, BBS excels in managing the more intricate structure of the spectra, achieving high accuracy with far fewer points compared to random sampling.

In this study, we intentionally limited the number of data points to 1200 to reflect realistic constraints in simulation-based workflows, where generating additional data through full-wave simulations like FDTD can be computationally prohibitive. The proposed augmented BBS method is specifically designed to be most effective during the early stages of data acquisition, providing improved prediction accuracy with fewer samples. Moreover, in practical Bragg grating applications, the achievable precision is often bounded by fabrication tolerances, meaning further increases in R^2^ beyond 0.95 may not translate into meaningful design improvements. Therefore, our focus remains on maximizing efficiency and performance within a limited data regime, which is more representative of real-world design constraints.

As the number of data points increases, the gap between the performance of BBS and random sampling narrows, reflecting the diminishing returns of additional data when the dataset nears saturation. However, BBS maintains its advantage by prioritizing the selection of the most informative data points. These findings highlight the capability of Bayesian BBS to optimize data acquisition in high-dimensional parameter spaces, reducing the required training dataset while maintaining or improving prediction accuracy.

### 4.2. Augmented BBS for Improved Database Generation

In the Bayesian approach, the Gaussian process regression (GPR) model selects data points by using the upper confidence bound (UCB) acquisition function, which identifies the data point with the highest predicted standard deviation from a dense mesh [49]. In our implementation of the UCB acquisition function, we set the weight parameter for the mean (μ) to zero and the standard deviation weight (β) to one, effectively prioritizing exploration over exploitation. In UCB, the mean term promotes exploitation (sampling where predictions are high), while the standard deviation term promotes exploration (sampling where the model is uncertain). By emphasizing the predictive uncertainty of the surrogate model, the acquisition function drives sampling toward underexplored areas. This maximizes the uncertainty in the model, leading to the acquisition of the most informative data point for further training of the GPR model.

However, due to the multi-dimensionality of the output space, the UCB acquisition function could have equal or almost equal values for several data points. This is more pronounced at the beginning of the data selection, when only a few data points are known. To improve the data selection procedure and avoid the limitations of having several data points with similar values for the acquisition function, we add an additional condition to the data selection procedure. This modified approach, augmented BBS, is as follows.

We first identify data points with maximum values for their acquisition function. This is accomplished using the predictive standard deviation for all the unknown data points. We use a small threshold epsilon (10^−6^) to account for numerical precision, capturing all values close to the maximum in the UCB acquisition function. If there are multiple candidates with the maximum acquisition value within the epsilon range, we take all these data points for the secondary screening. We then compute their Euclidean distances to all the points that have already been selected (selected data). We used Euclidean distance to assess point separation due to its simplicity and efficiency. All input parameters were normalized to the same range, making Euclidean distance an appropriate choice in the absence of strong inter-parameter correlations. While alternative distance metrics may be explored in future work, Euclidean distance provided a good balance between interpretability and computational cost in our setting. This returns a matrix of distances. We take the minimum distance for each candidate from the selected data. Among the candidates with the maximum acquisition value, we choose the data with the largest of these minimum distances, ensuring maximal separation. This guarantees that the selection process favors the points that not only have a high acquisition value but are also as diverse as possible–in other words, spread out within the design space.

**Figure 4:**
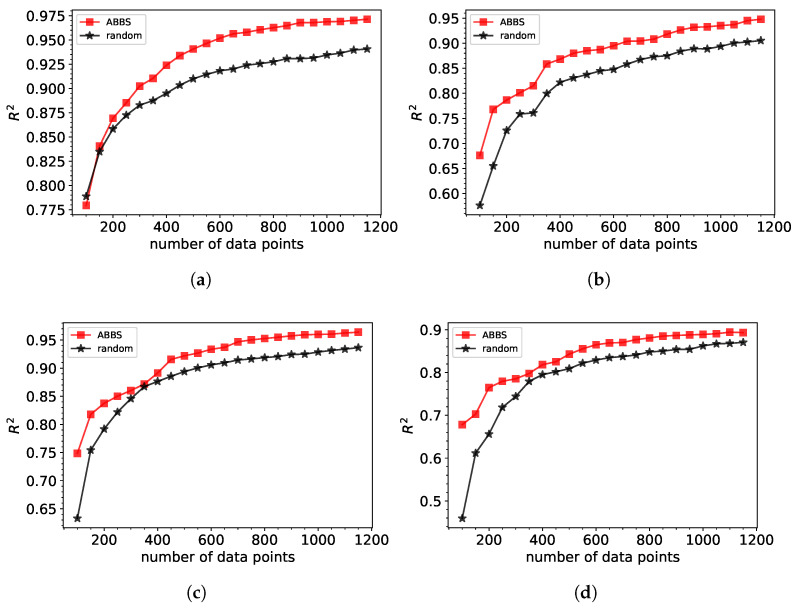
Performance of XGBoost machine learning model trained on database generated by augmented BBS (ABBS) approach and by a random selection of points for (**a**) Gaussian fit (**b**) polynomial of 3rd order (**c**) polynomial of 4th order (**d**) polynomial 5th order.

The augmented BBS method’s primary advantage lies in its ability to adaptively focus on regions of the parameter space that provide maximum informational gain, even as the dataset grows. By incorporating mechanisms to avoid redundancy and refine data selection based on prior iterations, the augmented BBS reduces the required training dataset. This is especially evident in Figure 4, where, in contrast to the standard, augmented BBS significantly outperforms random from the beginning of the database generation.

In addition to improving model accuracy with fewer training points, the augmented BBS approach also leads to a reduction in the training dataset acquisition time. Assuming all simulations require comparable computational effort, the smaller, more informative datasets generated by ABBS resulted in up to a 35% reduction in dataset acquisition compared to random sampling (for 4th- and 5th-order polynomial fits). This computational advantage is particularly relevant for iterative design workflows, where both simulation and training time are critical bottlenecks.

This early advantage of the augmented BBS method is attributed to the diversity criterion introduced via the epsilon threshold. At the start of database generation, the UCB acquisition function yields several candidates with nearly identical values due to the limited training data. The epsilon filter identifies such points, and the algorithm then selects the one farthest from existing data points. This ensures that the initial selections are not only informative in terms of uncertainty but also well-distributed across the parameter space, enabling faster learning and better generalization even with small datasets.

Overall, the augmented BBS method enhances the efficiency of Bayesian optimization by improving data selection strategies, making it particularly valuable for applications involving high-dimensional parameter spaces and resource-intensive simulations. It offers a practical pathway for achieving high prediction accuracy with minimal data, thereby reducing computational costs and enabling faster convergence in machine learning workflows.

To apply machine learning methods discussed in this work, we use two distinct models tailored to different stages of the methodology. For Bayesian data selection, we employ a Gaussian process regressor. This model provides both predictions and uncertainty estimates, which are essential for guiding the acquisition function. For the machine learning modeling of the Bragg grating response, we use XGBRegressor from the XGBoost library, chosen for its strong performance in structured regression tasks. Evaluation of model accuracy and generalization is carried out using standard regression metrics from the scikit-learn library.

## 5. Conclusions

In this study, we explored a Bayesian-based sampling (BBS) approach for the efficient generation of simulation data for Bragg grating structures—critical components in modern optical sensing technologies. The constructed database consisted of reflectance spectra corresponding to various geometrical configurations of the gratings. We analyzed the performance of different fitting functions applied to the central lobe of the reflectance spectrum, including polynomials of varying orders and Gaussian fits. Our results showed that polynomial fitting outperforms Gaussian fitting due to inherent asymmetries in the spectral shape, and we identified an optimal polynomial order that balances accuracy and complexity.

The BBS method has been shown to enhance data efficiency by selecting diverse and informative points within the design space. Notably, our findings highlight the importance of incorporating distance-based diversity in the early stages of database generation, particularly when dealing with complex and highly nonlinear spectral responses, conditions often encountered in real-world sensing applications.

Overall, this work provides practical insights into data-efficient modeling of Bragg gratings, offering a robust framework for the development of advanced machine learning-driven sensor design and optimization. These findings contribute to the broader effort to accelerate simulation-based design in photonic sensors, especially under constraints of limited computational resources or experimental data.

## Figures and Tables

**Table 1:** Precision of the fit to the Bragg reflectance for different functions. The minimum and the average values are calculated for the full database of available simulations. In the table *n* corresponds to the order of the polynomials.

Fitting Method	Fixed Bandwidth		Floating Bandwidth	
	av. $R^{2}$	av. RMSE	av. $R^{2}$	av. RMSE
Gaussian	0.9977	8.1 × 10^−4^	0.9976	1.1 × 10^−3^
Polynomial (n=3)	0.9864	1.6 × 10^−3^	0.9763	2.5 × 10^−3^
Polynomial (n=4)	0.9997	2.2 × 10^−4^	0.9998	3.0 × 10^−4^
Polynomial (n=5)	0.9999	8.7 × 10^−5^	0.9998	1.8 × 10^−5^

**Table 2:** Precision of the fit to the Bragg reflectance for different functions based on floating frequency range. The minimum and the average values are calculated for the full database of available simulations. In the table *n* corresponds to the order of the polynomials.

Fitting Method	Floating Bandwidth					
	av. $R^{2}$	st. $R^{2}$	av. RMSE	st. RMSE	av. MAPE	st. MAPE
Gaussian	0.9914	2.1 × 10^−3^	4.8 × 10^−5^	2.0 × 10^−6^	0.33	0.14
Polynomial (n=3)	0.9773	5.9 × 10^−3^	9.7 × 10^−5^	3.0 × 10^−5^	3.08	2.46
Polynomial (n=4)	0.9859	8.0 × 10^−3^	3.1 × 10^−3^	2.0 × 10^−3^	2.8	2.14
Polynomial (n=5)	0.9549	8.1 × 10^−3^	6.3 × 10^−3^	1.2 × 10^−3^	6.3	2.25

**Table 3:** Precision of parameters and curve prediction with XGBoost using the databases obtained by different fitting methods with floating bandwidth.

Fitting Method	Parameter Prediction		Curve Prediction	
	$R^{2}$	RMSE	$R^{2}$	RMSE
Gaussian	0.9914	4.8 × 10^−5^	0.9917	3.85 × 10^−6^
Polynomial (n=3)	0.9773	9.7 × 10^−5^	0.9648	6.49 × 10^−6^
Polynomial (n=4)	0.9859	3.1 × 10^−3^	0.9544	3.71 × 10^−6^
Polynomial (n=5)	0.9549	6.3 × 10^−3^	0.9515	1.79 × 10^−6^

## Data Availability

Data available in a publicly accessible repository https://github.com/NechepurenkoIgor/Data-Files-for-Augmented-Bayesian-Data-Selection (accessed on 3 August 2025).

## Abbreviations

The following abbreviations are used in this manuscript:

ABBS	Augmented Bayesian-based sampling
BBS	Bayesian-based sampling
BO	Bayesian Optimization
CMT	Coupled mode theory
GPR	Gaussian process regression
ML	Machine learning
RMSE	Root mean squared error
UCB	Upper confidence bound
XGBoost	Extreme gradient boosting
